# Introgressive hybridization in a trophically polymorphic cichlid

**DOI:** 10.1002/ece3.841

**Published:** 2013-10-18

**Authors:** C Darrin Hulsey, Francisco J García-de-León

**Affiliations:** 1 Department of Ecology and Evolutionary Biology, University of Tennessee569 Dabney Hall, Knoxville, Tennessee, 37996; 2 Laboratorio de Genética para la Conservación, Centro de Investigaciones Biologicas del Noroeste, Instituto Politécnico Nacional 195 Playa Palo de Santa Rita SurLa Paz, B.C.S. México 23096

**Keywords:** Gene flow, Mexico, pharyngeal jaw, trophic adaptation

## Abstract

Trophically polymorphic species could represent lineages that are rapidly diverging along an ecological axis or could phenotypically mark the collapse of species through introgressive hybridization. We investigated patterns of introgression between the trophically polymorphic cichlid fish *Herichthys minckleyi* and its relative *H. cyanoguttatus* using a combination of population genetics and species tree analyses. We first examined the distribution of mitochondrial haplotypes within the alternative *H. minckleyi* pharyngeal jaw morphotypes that are endemic to the small desert valley of Cuatro Ciénegas. We recovered two clusters of mitochondrial haplotypes. The first contained a number of slightly differentiated cytochrome b (cyt*b*) haplotypes that showed some phylogeographic signal and were present in both jaw morphotypes. The other haplotype was monomorphic, highly differentiated from the other cluster, present in equal frequencies in the morphotypes, and identical to *H. cyanoguttatus* haplotypes found outside Cuatro Ciénegas. Then, we investigated whether *H. minckleyi* individuals with the *H. cyanoguttatus* cyt*b* were more evolutionarily similar to *H. cyanoguttatus* or other *H. minckleyi* using a species tree analysis of 84 nuclear loci. Both *H. minckleyi* pharyngeal morphotypes, regardless of their cyt*b* haplotype, were quite distinct from *H. cyanoguttatus*. However, hybridization could be blurring subdivision within *H. minckleyi* as the alternative jaw morphotypes were not genetically distinct from one another. Accounting for introgression from *H. cyanoguttatus* will be essential to understand the evolution of the trophically polymorphic cichlid *H. minckleyi*.

## Introduction

Trophic polymorphism can represent a transient intermediate stage in the divergence of lineages (Smith and Skulason 1996; Barluenga et al. 2006). For example, cichlid fish that are polymorphic in their jaw morphology could be in the process of ecological speciation (Meyer 1990). Yet, polymorphic species could also contain equally fit alternatives maintained through balancing selection (Nakajima et al. 2004; Hulsey et al. 2005). Alternatively, trophic variants that currently occur within the same population could also phenotypically characterize hybridizing lineages that are in the process of collapsing into a single entity (Daniels and Corbett 2003; Riley et al. 2003; Taylor et al. 2006). In part, hybridization's role in maintaining phenotypic diversity is receiving renewed attention because of the increasing availability of genetic markers that has made introgression easier to document (Moore 1995; Streelman et al. 2004; Mims et al. 2010). Furthermore, it has become clear that hybridization could also play an important role in the generation of phenotypic novelty (Rieseberg et al. 1999; Seehausen 2004; Parnell et al. 2012). The integration of more extensive genetic markers with new analytical tools will be required to unravel the evolutionary role of hybridization in maintaining and generating divergence in trophically polymorphic species. As a foundation for delineating among several alternative processes in a strikingly variable cichlid species, we used a combination of population genetics and species tree analyses to determine whether introgressive hybridization has occurred in the trophically polymorphic cichlid fish *Herichthys minckleyi*.

Hybridization can influence animal diversification (Dowling and Secor 1997; Seehausen 2004; Mallet 2005), and interspecific gene flow might be exceptionally important to the evolutionary divergence of narrowly endemic species (López-Pujol et al. 2012; Toews and Brelsford 2012). This geographic asymmetry in hybridization's importance might frequently be due to factors such as divergence in population size, adaptive divergence, and the frequency of genetic bottlenecks (Funk and Omland 2003; Chan and Levin 2005; Hudson et al. 2011). For instance, introgression might be favorable to species with small populations or restricted ranges because it could serve as a source of both novel genetic variation and subsequent phenotypic adaptation (Streelman et al. 2004; Tobler and Carson 2010). Yet, high rates of gene flow into endemic species from more widespread relatives could also swamp the gene pool of unique lineages and eliminate adaptive divergence (Hedrick 2005; Mallet 2005). Furthermore, although hybridization is likely important in species-rich communities (Seehausen 2004; Grant and Grant 2008), the collapse of interspecific reproductive barriers might be more likely in species-poor assemblages that inhabit small and geographically isolated regions (Riley et al. 2003; Carson and Dowling 2006). Hybridization's influence on phenotypic novelty within these small assemblages might also be most easily detected when introgression results in phenotypic variation that is exhibited as discrete alternative morphotypes (Daniels and Corbett 2003; Pardo-Diaz et al. 2012).

**Figure 1:**
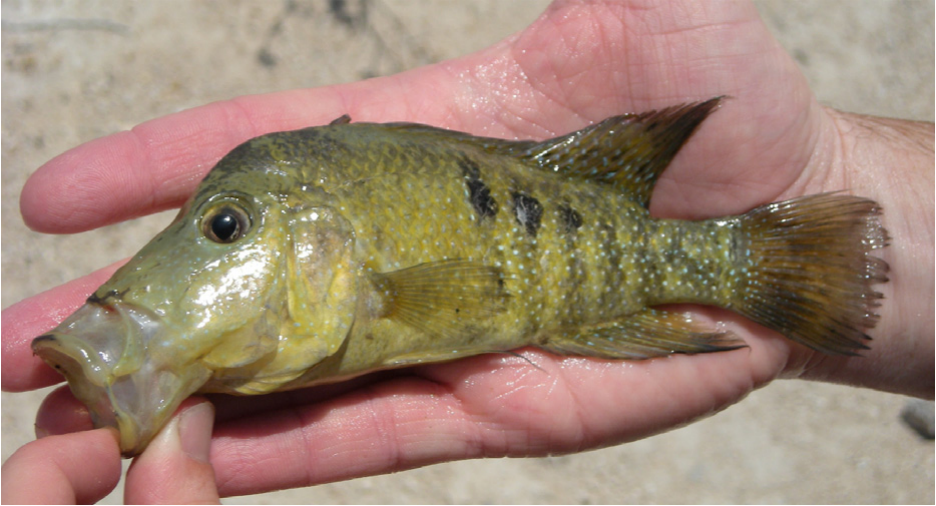
*Herichthys minckleyi,* which is completely endemic to Cuatro Ciénegas, displaying its oral jaws.

The cichlid fish *Herichthys minckleyi* (Fig. 1) is bimodal in several aspects of its trophic morphology (Kornfield and Koehn 1975; Trapani 2003; Hulsey et al. 2005; Hulsey 2006). Generally, pharyngeal tooth size has been used to divide *H. minckleyi* into one of two morphotypes (Fig. 3A,B). “Molariforms” have enlarged molar-like teeth and “papilliform” pharyngeal jaws exhibit only small pointed teeth (Kornfield and Taylor 1983). The pharyngeal jaw variation in *H. minckleyi* is also associated with feeding differences (Hulsey et al. 2005). This type of largely discrete phenotypic difference might readily lead to the inference that the morphotypes should be diagnosed as two distinct species diverging along an ecological axis. However, the two pharyngeal morphotypes have been observed to interbreed in the wild (Kornfield et al. 1982), consistently occur together in sympatry (Hulsey et al. 2008), and are indistinguishable at a number of allozyme loci (Kornfield and Koehn 1975; Sage and Selander 1975; Kornfield et al. 1982). Nevertheless, more extensive genetic analyses could improve the fine-scale understanding of the distinctiveness of cichlid lineages in the unique desert valley where *Herichthys minckleyi* is endemic.

**Figure 2:**
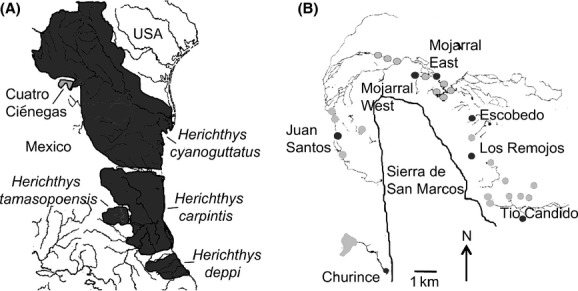
The *Herichthys* species ranges (A). The collection sites of *Herichthy minckleyi* from Cuatro Ciénegas (B) are depicted.

The geography of *Herichthys* lineages in Northeastern Mexico provides an exceptionally tractable setting to test alternative patterns of divergence and gene flow (Fig. 2). *Herichthys minckleyi* is only found in a small (∼1500 km^2^) valley called Cuatro Ciénegas located in the center of the Mexican Chihuahuan desert (Minckley 1969). Within this valley, both pharyngeal morphotypes of *Herichthys minckleyi* always co-occur in a series of approximately 200 spring-fed streams and pools. These aquatic habitats are embedded within a desert matrix and generally have infrequent surface connections (Chaves-Campos et al. 2011a). *Herichthys minckleyi's* sister group contains four species (*H. cyanoguttatus, H. carpintis*, *H. tamasopoensis*, and *H. deppii*) that are approximately two million years divergent from *H. minckleyi* (Hulsey et al. 2004, 2006), but *H. cyanoguttatus* at least retains the capacity to make fertile hybrids with *H. minckleyi* in the lab (C. D. Hulsey, unpublished data). As *H. minckleyi* and *H. cyanoguttatus* are the two most northern cichlids in the Neotropics and because *H. cyanoguttatus* is the only cichlid whose range currently overlaps with *H. minckleyi* in a small lobe of the Cuatro Ciénegas basin (Minckley 1969; Miller et al. 2006), recent gene flow between *H. minckleyi* and a cichlid other than *H. cyanoguttatus* is unlikely.

Mitochondrial sequences have long been used to resolve relationships among closely related animal species (Moore 1995; Dowling and Secor 1997; Hulsey et al. 2013). Mitochondrial sequences might therefore provide a useful initial marker to examine genetic differentiation among *Herichthys* species as well as the two *H. minckleyi* pharyngeal morphotypes. Mitochondrial divergence could also provide testable inferences concerning the phylogeography of *H. minckleyi* because the mitochondrial differentiation is extensive among other Cuatro Ciénegas endemics including snails, fish, shrimp, and turtles (Johnson 2005; Carson and Dowling 2006; Chaves-Campos et al. 2011a,b; McGaugh 2012). Mitochondrial divergence in these other species has also highlighted the potential for gene flow between a tributary of the Rio Grande and Cuatro Ciénegas via several man-made canals that now connect these two hydrologically distinct basins (Chaves-Campos et al. 2011b). Because of the substantial divergence (>4%) between the mitochondrial haplotypes of *H. minckleyi* and *H. cyanoguttatus* (Hulsey et al. 2004), mitochondrial sequences could also provide a useful indicator of the extent and timing for any gene flow between these two *Herichthys* species.

However, the inferences that can be drawn from mitochondrial markers alone are limited (Arnold 1993). Because of the maternal inheritance and nonrecombining nature of mitochondria, these markers could easily generate misleading discontinuities via random processes (Irwin 2002; Toews and Brelsford 2012). Mitochondrial gene trees can also be quite discordant from the nuclear genome because of more deterministic processes (Funk and Omland 2003; Chan and Levin 2005). Sex-biased dispersal, asymmetric breakdown in species recognition systems, and molecular adaptation can all lead to biases in the rate of mitochondrial introgression (Doiron et al. 2002; Bossu and Near 2009; Chatfield et al. 2010; Singhai and Moritz 2012). However, incongruence among individual gene trees and the containing species tree, or incomplete lineage sorting (ILS), is generally widespread among all loci and can be accounted for when a large number of independent sequence markers are available to infer evolutionary processes (Maddison and Knowles 2006; Corl and Ellegren 2013). Yet, ILS can result from several processes including the retention of ancestral polymorphism as well as introgression of genes through interspecific hybridization (Maddison 1997; Hudson et al. 2011). Fortunately, there are a number of new coalescent-based methods that account for ILS among gene trees, and despite the mosaic nature of most lineages, these methods can provide robust inferences of the true species tree. For instance, with a large number of markers, a species tree analysis could test whether the alternative pharyngeal morphotypes within *H. minckleyi* should be considered distinct lineages.

Species tree approaches could also highlight cases of introgressive hybridization among markers. Although many coalescent-based approaches assume introgressive hybridization between species is not a source of ILS (Maddison and Knowles 2006; Knowles 2009), some methods are agnostic as to the sources of gene tree heterogeneity (Chung and Ané 2011). If hybridization has structured the relationships among *Herichthys* lineages, it might be best to estimate their containing species tree without the underlying assumption that ancestral polymorphism has provided the primary source of gene tree incongruence (Kubatko 2009). Species tree reconstruction that used a large number of nuclear markers could therefore offer a powerful means to estimate the dominant evolutionary relationships among the two taxonomically diagnosed *Herichthys* and alternative morphotypes present in Cuatro Ciénegas. Furthermore, a species tree based on a substantial sampling of the nuclear genome could determine whether introgression of mitochondria is likely occurring in the polymorphic *H. minckleyi*.

Using a series of population genetic and species tree analyses, we examined introgression and lineage differentiation within the cichlids of Cuatro Ciénegas. We first examined the distribution of mitochondrial haplotypes within *H. minckleyi* and its closely related *Herichthys* species. Then, we asked whether there was evidence that particular haplotypes were more common in either the papilliform or molariform *H. minckleyi*. Using a subset of individuals, we also employed a gene tree/species tree analysis of 84 nuclear loci to ask whether *H. minckleyi* individuals with *H. cyanoguttatus* mitochondrial haplotypes were more similar in their nuclear loci to *H. cyanoguttatus*. We used this same framework as well as a reduced set of nuclear loci to assess whether there was evidence for a clear containing tree for either of the jaw morphotypes within *H. minckleyi*.

## Methods

### Field collections

*Herichthys minckleyi* were collected over several trips to Cuatro Ciénegas between 2000 and 2008. To highlight the overall morphological bimodality of the pharyngeal jaw morphology in *H. minckleyi*, we quantified the tooth area of 160 specimens. Using preserved specimens genotyped below as well as other fish collected from Cuatro Ciénegas, we first dissected out their lower pharyngeal jaws. These bony elements were cleaned of all muscle and fascia and allowed to dry. Then, we took a digital image of the jaws with a ruler in frame and imported it into the program ImageJ (Schneider et al. 2012). The dorsal area of the posterior-most tooth along the right of the suture that divides the pharyngeal jaw (Hulsey 2006) was then quantified. We also measured standard length, the distance from the upper jaw tip to the caudal peduncle, with dial calipers on each specimen to control for body size. Tooth areas were square-root-transformed to linearize the measurements, and then, the residuals of these measurements and standard length were obtained using reduced major axis regression. The residuals were then plotted in 25 bins, and the frequency of tooth area residuals within these bins was used to highlight the bimodality of *H. minckleyi*'s pharyngeal morphology.

The cichlids genotyped were limited to individuals that could be unambiguously assigned to either the molariform or papilliform morphotype in the field following methods used in previous studies (Kornfield and Taylor 1983; Hulsey et al. 2005). The pharyngeal phenotypes of *H. minckelyi* were assessed using an otoscope placed into the oral jaws of the fish. If enlarged molariform teeth were present, the individual was classified as molariform. If only small pointed teeth were present on the jaw, the individuals were classified as papilliform. Small individuals (<70 mm) and the relatively rare (<5%) proportion of *H. minckleyi* with difficult-to-diagnose pharyngeal morphology were not included in our analyses. We reasoned that excluding these individuals was warranted because these small and uncommon intermediate individuals could obfuscate any patterns of genetic differentiation that might exist between the generally bimodal *H. minckleyi* pharyngeal phenotypes. In total, 90 molariform and 95 papilliform *H. minckleyi* were examined from Cuatro Ciénegas. Other species used in the analyses were collected from their native range from sites in Hulsey et al. (2004).

### Primer design and sequencing

For sequencing, genomic DNA was isolated from fin clips. The entire cytochrome *b* (cyt*b*) gene was PCR-amplified using primers in Hulsey et al. (2004). We then utilized the ProR1 primer to sequence 563 base pairs (bp) of the cyt*b* gene for the 185 *H. minckleyi*. We combined this with sequence data available for cyt*b* for *H. carpintis*, *H. tamasopoenis*, *H. deppii*, and *H. cyanoguttatus* from Hulsey et al. (2004) that represent the same individuals sequenced for nuclear loci below. We also sequenced an additional ten *H. cyanoguttatus* from several parts of its native range to compare with any putative *H. cyanoguttatus* sequences recovered from *H. minckleyi* in Cuatro Ciénegas. These sequences have been submitted to GenBank (KC839618-KC839812).

Replicating the number of individuals sampled frequently can provide more power to differentiate among alternative hypotheses. However, substantial replication in the number of independent nuclear genes sampled can also provide substantial power to examine patterns such as mitochondrial introgression and genetic differentiation of morphotypes (Sunnucks 2000; Jennings and Edwards 2005; Corl and Ellegren 2013). Therefore, we chose to examine a limited set of *Herichthys* individuals with a robust sampling of nuclear loci within a species tree framework. We sequenced our set of nuclear loci for a single individual of *H. carpintis*, *H. tamasopoenis*, *H. deppii* and *H. cyanoguttatus*, and four *H. minckleyi* collected from the wild in Mexico. The *H. carpintis* was collected from Laguna Champayan in Tamaulipas, *H. tamasopoenis* from Rio Tamasopo in San Luis Potosi, *H. deppii* from the Rio Nautla in Veracruz, and *H. cyanoguttatus* from the Rio San Fernando in Tamaulipas. The four *H. minckleyi* were collected on the same date from the Churince site and were chosen to represent combinations of pharyngeal jaw morphology and genotyped cyt*b* haplotypes. We used one molariform and one papilliform with the “*H. cyanoguttatus*” cyt*b* haplotype and one molariform and one papilliform with the identical “*H. minckleyi*” haplotype.

To obtain loci for the gene tree/species tree analyses, we used primers obtained in two ways. First, we screened a large number of published primers for cichlids (Carleton and Kocher 2001; Mims et al. 2010; Hulsey et al. 2011). To generate sequences from additional loci, we purchased a sequence library of 400 randomly cloned and amplified loci that were each approximately 700 bps (Lucigen Corporation, Madison, Wisconsin). Sequences for these loci were obtained from genomic DNA of one *H. cyanoguttatus*. We narrowed these sequences to 150 high-quality reads that were loaded into Primer 3 v. 0.4.0 (Rozen and Skaletsky 2000) and used to generate forward and reverse primers approximately 27 bp in length. Each primer set was designed to produce an approximately 500 bp amplicon. The final set of primers (Table S1) were utilized because they amplified PCR product, generated a single electrophoretic gel band, sequenced with the forward primer, and exhibited at least one mutation among the individuals analyzed.

Amplifications were carried out in an Eppendorf DNA thermocycler. Thermal cycling conditions for all loci consisted of an initial denaturation step of 94°C (30 s), an annealing step of either 55°C or 49°C (30 s) and an extension of 72°C (1.5 min). A final incubation of 72°C for 5 min was added to the end of reactions to ensure complete extension of products. Subsequently, the PCR products were electrophoretically checked for amplification of a single band using an agarose gel with ethidium bromide (1 mg/L) added and run in Tris–acetate buffer (pH 7.8). Positively amplified DNA was purified using an enzymatic combination of 1 μL of Exonuclease I and 1 μL shrimp alkaline phosphatase per 10 μL of PCR product. PCR products were sequenced at the High-Throughput DNA Sequencing Facility at the University of Washington using traditional Sanger sequencing. Sequences were assembled using the program Sequencher version 4.1(Gene Codes, Ann Arbor, Michigan). For analyses, sequences were aligned using Clustal X (Larkin et al. 2007). All nuclear sequences are on GenBank (Table S2).

### Mitochondrial haplotype analyses

We initially inspected *H. minckleyi* mitochondrial haplotype divergence as a haplotype network. In this analysis, all of the Cuatro Ciénegas haplotypes were combined with mitochondrial sequences available from the four closely related species *H. tamasopoensis*, *H. deppii*, *H. carpintis*, and *H. cyanoguttatus*. The haplotype network was constructed using parsimony as implemented in TCS 1.21 (Clement et al. 2000), and the haplotype frequency in each haplogroup was tallied. Finally, because “*H. cyanoguttatus*” haplotypes were obtained from fish that were morphologically identified as *H. minckleyi* molariforms and papilliforms, we examined the cyt*b* differentiation further. We performed a chi-square test with the null expectation of molariforms and papilliforms exhibiting equal proportions of the “*H. cyanoguttatus*” cyt*b* haplotype. A significant deviation from equal proportions of this haplotype would indicate the “*H. cyanoguttatus”* haplotype was more common in either the molariform or papilliform morphotype.

### Nuclear species tree reconstruction

Using the program jModeltest 2 (Darriba et al. 2012), the best DNA substitution models for each locus were inferred using the Akaike information criterion. Gene trees were estimated using MrBayes version 3.2 (Ronquist et al. 2012). For each locus, we obtained a set of trees from two Bayesian iterations of five million Markov chain Monte Carlo (MCMC) generations with a sample frequency of 1000 and a burn-in period of two million generations. The remaining post-burn-in gene trees from a pair of MrBayes runs for each locus were used as input for the species tree analysis. We also examined the effective sample size of the likelihoods of each gene tree analysis remaining post-burn-in using Tracer v1.5 (Drummond and Rambaut 2007) to ensure values were over 200 and the MCMC chains converged to a stationary distribution.

To estimate the species tree, we used the multilocus method BUCKy that applies a Bayesian approach to reconstruct the dominant gene tree history for a set of taxa (Ané et al. 2007). Instead of modeling ILS and the multispecies coalescent specifically, BUCKy summarizes the posterior distributions of individual gene trees and identifies the dominant branching patterns for a given group of taxa across all of the individual gene tree distributions. This containing tree is comprised of those clades whose concordance factors (CF), defined as the proportion of the genome for which a given clade is true, exceed those of other contradictory clades (Ané et al. 2007). For the analyses, we used the subprogram “mbsum” to summarize the MrBayes produced gene tree distributions for each locus and to perform Bayesian concordance analysis to generate the species tree. In BUCKy, the dominant species tree was obtained from 5 million MCMC cycles with four heating chains. We originally ran BUCKy for all 84 nuclear loci for the eight *Herichthys* individuals.

The retention of ancestral nuclear alleles and/or introgression of alleles from *H. cyanoguttatus* could heavily influence the concordance factors above and our inferences concerning the two pharyngeal morphotypes in *H. minckleyi*. Therefore, we ran an additional BUCKy analysis that pruned all the loci that contained identical alleles in *H. minckleyi* and *H. cyanoguttatus*. Every locus that showed no sequence divergence between *H. cyanoguttatus* and any of the four *H. minckleyi* were removed. A BUCKy analysis that mirrored methods detailed above for the full nuclear data set was then run on the remaining nuclear loci. The resulting CF values from both the full and reduced loci analyses were then depicted on a subset of the possible topological relationships for the sequenced individuals.

## Results

### Tooth area morphometrics

**Figure 3:**
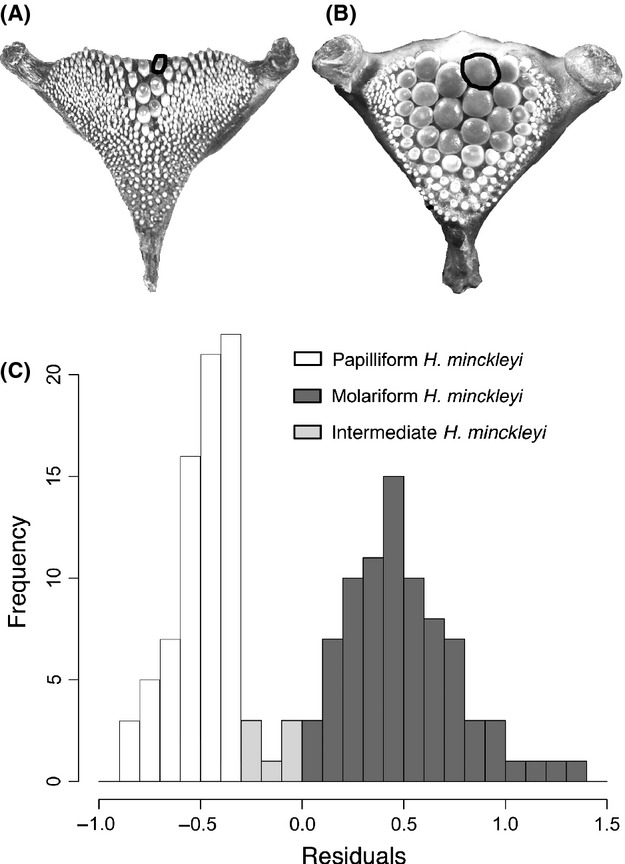
The lower pharyngeal jaw of papilliform (A) and molariform (B) *Herichthys minckleyi*. The presence of large flattened molar-like teeth characterizes molariforms. The absence of these molar-like teeth and the presence of only small pointed teeth diagnose papilliforms. The pharyngeal tooth size of *H. minckleyi* (C) is bimodal and individuals with intermediate pharyngeal morphology are rare.

The tooth areas within *H. minckleyi* are highly bimodal in their distribution and individuals with intermediate tooth morphologies are relatively rare (Fig. 3C).

### Mitochondrial haplotype analyses

**Figure 4:**
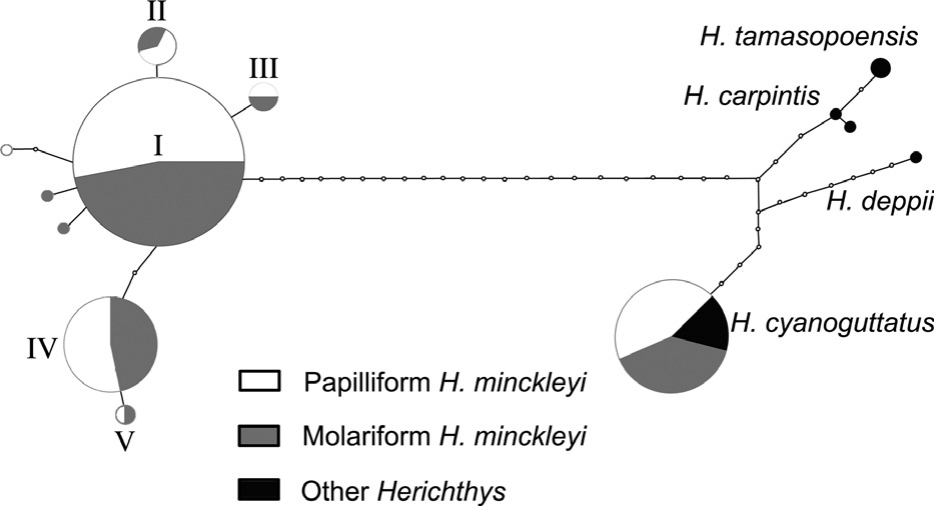
Cyt*b* haplotype divergence among the *Herichthys* depicted using a parsimony haplotype network. Circle size approximates haplotype frequencies. Major groups exhibiting “*H. minckleyi*” mitochondrial haplotypes are labeled I-V. The other *Herichthys* species that include the “*H. cyanoguttatus*” haplotype found in Cuatro Ciénegas are quite divergent from the “*H. minckleyi”* haplotypes. The proportion papilliform *H. minckley* representing each haplotype group is colored white and molariform representation is shaded gray. All five common “*H. minckleyi*” haplotypes are found in both molariforms and papilliforms. Likewise, the “*H. cyanoguttatus*” haplotypes are found in both molariforms and papilliforms in roughly equal frequencies.

The mitochondrial analyses produced several interesting patterns (Table 1; Fig. 4). First, there were two clusters of cyt*b* haplotypes present in Cuatro Ciénegas. These two haplotype clusters were over 4% divergent and there was a noticeable lack of intermediate haplotypes. One of the haplotype groups, hereafter “*H. minckleyi*” haplotypes, exhibited substantial variation and some phylogeographic structure within the basin. The other haplotype (hereafter “*H. cyanoguttatus*”) was identical to the haplotype present in *H. cyanoguttatus* outside the Cuatro Ciénegas basin. Second, the two *H. minckleyi* pharyngeal morphotypes contained nearly the same ratio of “*H. cyanoguttatus*” cyt*b* haplotypes (*X* = 0.291; *P* = 0.59). Of the 90 molariforms, 29 possessed the “*H. cyanoguttatus*” haplotype. Of the 95 papilliforms, a total of 26 exhibited the “*H. cyanoguttatus*” haplotype. Third, *H. minckleyi* from Juan Santos in the western part of the valley contained the highest proportion of “*H. cyanoguttatus*” haplotypes (80%), while the isolated pool Escobedo in the eastern part of the valley contained the lowest proportion (<4%). Finally, all locations sampled within Cuatro Ciénegas except Los Remojos (*n* = 2) contained this “*H. cyanoguttatus”* cyt*b* haplotype (Table 1).

**Table 1:** Cuatro Ciénegas sampling locations, their GPS coordinates, the number of *Herichthys minckleyi* sampled, and the number of molariforms (M) and papilliforms (P) examined at each location are given. Individuals are divided into major cyt*b* haplotypes (C = *H. cyanoguttatus* and haplotypes I–V = *H. minckleyi* groups in Fig. 4)

				Major Haplotype Groups					
				
Location	GPS Coordinates		Number of *Herichthys minckleyi* (M/P)	C	I	II	III	IV	V
Churince	26° 50.53N	102° 08.20W	19 (9/10)	2	8	0	9	0	0
Juan Santos	26° 53.86N	102° 08.81W	40 (27/13)	32	7	0	0	1	0
Mojarral West	26° 55.47N	102° 07.50W	72 (34/38)	11	49	9	0	3	0
Mojarral East	26° 55.48N	102° 07.28W	18 (8/10)	5	8	1	1	3	0
Los Remojos	26° 55.01N	102° 06.67W	2 (1/1)	0	0	1	0	1	0
Escobedo	26° 52.30N	102° 05.26W	29 (10/19)	1	4	0	0	24	0
Tio Candido	26° 52.33N	102° 04.85W	6 (2/4)	3	0	0	0	1	2

There were also some interesting geographic patterns in the distribution of “*H. minckleyi*” cyt*b* haplotypes. All of the “*H. minckleyi”* haplotypes that were not unique to a single individual were recovered from both molariform and papilliform *H. minckleyi* (Fig. 4; Table 1). Furthermore, most of the “*H. minckleyi”* haplotypes were not unique to a single location. Haplotype I was the most common cyt*b* sequence and was present in every location but Tio Candido and Los Remojos. However, the absence of this haplotype could be due to the low sample size from these locations. Hapotype II was most common at Mojarral West and was also recovered from Mojarral East and Tio Candido. Haplotype III was most common at Churince but one individual in Mojarral East contained this haplotype. Haplotype IV was present at low frequency in every location except Churince, but it was exceptionally abundant in Escobedo. Haplotype V was restricted to Tio Candido.

### Nuclear species tree reconstruction

Our original 400 anonymous sequences were narrowed to 84 variable nuclear loci. When Sanger sequences of these loci were aligned, they produced a total of 38,933 bps of sequence for each of the eight *Herichthys* individuals (Table S1). A median number of 447 bp were aligned per locus. The median value for maximum sequence divergence of the 84 loci among all the *Herichthys* was 0.7 ± 0.6%. Despite the large mitochondrial divergence (4.8%) within *H. minckleyi* (Table 2), average nuclear sequence divergence among the four *H. minckleyi* was minute (0.1%) compared to their consistently higher percent sequence divergence from both *H. cyanoguttatus* and the other *Herichthys* species (∼0.5%).

**Table 2:** Uncorrected pairwise divergences of the eight *Herichthys*. As denoted with a superscript, the molariform (M) and papilliform (P) *Herichthys minckleyi* either exhibited haplotype III from the Churince collection site or the “*H. cyanoguttatus*” (C) mitochondrial haplotype. Individuals are the same on both axes. Names are abbreviated on the *x*-axis. Above the diagonal, the average uncorrected pairwise divergence for all 84 nuclear loci is depicted. Below the diagonal, divergence in the cyt*b* haplotypes is given. Two *H. minckleyi*, like the *H. cyanoguttatus*, exhibit 4.8% divergence at cyt*b* from the other *H. minckleyi*. In the nuclear loci, all four of the *H. minckleyi* show little average divergence, but all have a 0.4% mean nuclear divergence from *H. cyanoguttatus*

	P^III^	M^C^	P^C^	M^III^	C	D	T	R
*Herichthys minckleyi* (P^III^)	*	0.0	0.0	0.1	0.4	0.6	0.5	0.5
*H. minckleyi* (M^C^)	4.8	*	0.0	0.0	0.4	0.6	0.5	0.4
*H. minckleyi* (P^C^)	4.8	0.0	*	0.0	0.4	0.6	0.4	0.4
*H. minckleyi* (M^III^)	0.0	4.8	4.8	*	0.4	0.6	0.4	0.4
*H. cyanoguttatus* (C)	4.8	0.0	0.0	4.8	*	0.3	0.2	0.2
*H. deppii* (D)	2.1	2.1	2.1	5.3	2.3	*	0.3	0.3
*H. tamasopoensis* (T)	2.0	2.0	2.0	5.0	2.0	2.1	*	0.2
*H. carpintes* (R)	4.8	1.8	1.8	4.8	0.5	2.1	1.8	*

**Figure 5:**
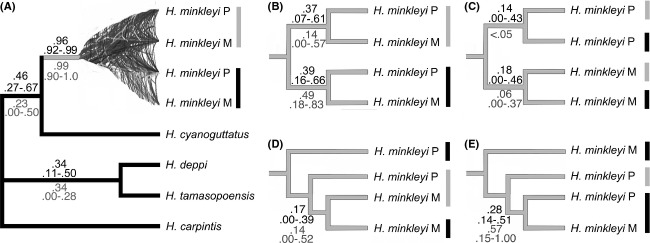
The BUCKy generated species trees for the eight *Herichthys*. The concordance factors (CF) and their 95% confidence intervals (CI) for the analysis of 84 nuclear loci are shown in black along the branches leading to a node. The CF and 95% CI for the 45 loci are given in gray. The dominant topology of the *Herichthys* species is shown in black and the tangle of *H. minckleyi* gene trees that do not conform to a single containing tree is highlighted (A). Despite two of the *H. minckleyi* exhibiting identical *H. cyanoguttatus* cyt*b* haplotypes, *H. cyanoguttatus* and the other *Herichthys* are genetically quite distinct from *H. minckleyi* in their nuclear genes. Additionally, the molariform (M) and papilliform (P) *H. minckleyi* with “*H. minckleyi”* cyt*b* haplotypes (gray bar) and the molariform (M) and papilliform (P) *H. minckleyi* with “*H. cyanoguttatus”* cyt*b* haplotypes (black bar) are monophyletic (concordance factor = 0.96). Four of the 15 possible rooted species tree topologies for the *H. minckleyi* are depicted (B-E). The CF values do not support a distinct containing tree for either the molariform or papilliform *H. minckleyi*.

The CF values of both BUCKy analyses were depicted on a subset of the possible phylogenetic relationships among the eight *Herichthys* (Fig. 5). The grouping of *H. cyanoguttatus* with *H. minckleyi* received a CF of 0.46 indicating *H. cyanoguttatus* shares more nuclear gene tree similarity with *H. minckleyi* than it does with the other *Herichthys* examined. However, the 84 nuclear genes did not recover the molariform and papilliform *H. minckleyi* with “*H. cyanoguttatus”* cyt*b* as grouping with the *H. cyanoguttatus* from outside the valley. Instead, the monophyly of all four *H. minckleyi* (Fig. 5A) was supported with a 0.96 CF (95% credibility: 0.92–0.99). The depicted subset of the 15 possible rooted relationships for the four *H. minckleyi* individuals highlight the general overlapping 95% CF credibility intervals (Fig. 5–B–E) for the possible bifurcating relationships among the *H. minckleyi*. Interestingly, the highest concordance factors within *H. minckleyi* (Fig. 4B) supported the two individuals with the “*H. minckleyi*” cyt*b* haplotype as a group with a 0.39 CF (95% credibility: 0.16–0.66) and the two individuals with the “*H. cyanoguttatus*” cyt*b* haplotype as another group with a 0.37 CF (95% credibility: 0.07–0.61). The pairs of pharyngeal morphotypes did not receive substantial CF support (Fig. 5C).

Of the 84 nuclear loci, 23 were monomorphic in the four *H. minckleyi* as well as *H. cyanoguttatus*. An additional 16 loci showed no sequence differences between at least one of the *H. minckleyi* and *H. cyanoguttatus*. The second BUCKy analysis run with the remaining 45 nuclear loci did not fundamentally change our inferences (Fig. 5). Similar to the 84 loci analysis, the molariform and papilliform with the “*H. cyanoguttatus*” cyt*b* haplotype grouped (Fig. 5B) with a 0.49 CF (95% credibility: 0.18–0.83). The major difference observed between this 45 loci analysis and the 84 loci analysis (Fig. 5E) was that the papilliform with the “*H. minckleyi*” cyt*b* haplotype grouped with the two individuals that had the “*H. cyanoguttatus*” cyt*b* haplotype (0.57 CF; 95% credibility: 0.15–1.00). Neither the analysis of 84 loci nor of 45 loci provided support for the two pharyngeal morphotypes forming genetically distinct lineages.

## Discussion

The mitochondrial divergence within *H. minckleyi* provides several insights into the distinctiveness of cichlid lineages within Cuatro Ciénegas. First, there are multiple endemic cyt*b* haplotypes within these fish, but these haplotypes are common to individuals exhibiting both molariform and papilliform pharyngeal morphology. This mitochondrial sharing among the pharyngeal morphotypes lends support to previous studies suggesting the two *H. minckleyi* morphotypes should be considered a single evolutionary lineage (Kornfield and Koehn 1975; Sage and Selander 1975; Kornfield et al. 1982). But, the presence of “*H. cyanoguttatus”* haplotypes in both pharyngeal morphotypes in multiple distinct locations within the Cuatro Ciénegas valley also indicates that *H. minckleyi* likely contains introgressed mitochondria from its close relative *H. cyanoguttatus*. It is feasible that the “*H. cyanoguttatus”* haplotype within *H. minckleyi* represents the retention of ancestral mitochondrial polymorphism (Maddison 1997; Hudson et al. 2011; Hulsey et al. 2011). However, the extensive sequence divergence (>4%) of the cyt*b* clusters, the lack of intermediate haplotypes within *H. minckleyi*, and the presence of this exact haplotype within *H. cyanoguttatus* outside the valley all argue that the presence of “*H. cyanoguttatus”* cyt*b* sequences within *H. minckleyi* is the result of recent introgression.

The lack of genetic variation in the isomorphic “*H. cyanoguttatus”* haplotype contrasts strongly with the divergence in the “*H. minckleyi”* cyt*b* haplotypes. The variation in the “*H. minckleyi*” haplotype cluster indicates that there could be some geographically structured divergence in *H. minckleyi* populations. For instance, a few of the “*H. minckleyi”* haplotype groups were found primarily in one collection location (Table 1). Phylogeographic structure in this endemic cichlid is not surprising because it mirrors patterns found in other aquatic organisms that are endemic to the Cuatro Ciénegas valley (Johnson 2005; Carson and Dowling 2006; Chaves-Campos et al. 2011a,b; McGaugh 2012). However, the extreme bimodality in cyt*b* sequences makes many phylogeographic inferences concerning *H. minckleyi* populations questionable. It seems feasible that sequencing cyt*b* for a large number of additional *H. minckleyi* could provide the power to make inferences about the phylogeographic structure of this single locus if “*H. cyanoguttatus*” haplotypes were filtered from a larger data set. Yet, in general, genetically informed conservation decisions about the unique and endangered *H. minckleyi* will likely become increasingly obscured if hybridization with *H. cyanoguttatus* continues (Riley et al. 2003).

In contrast to our mitochondrial inferences concerning the distinctiveness of *H. minckleyi* from *H. cyanoguttatus*, the containing species tree derived from nuclear loci overwhelmingly supports *H. minckleyi* monophyly (Fig. 5). The molariform and the papilliform Cuatro Ciénegas cichlids that contain the “*H. cyanoguttatus”* mitochondrial haplotype are genetically much more similar to other *H. minckleyi* than to the *H. cyanoguttatus* (Fig. 5). This discordance between the mitochondrial and nuclear markers lends support to the inference that there has been mitochondrial introgression from *H. cyanoguttatus* into *H. minckleyi* (Chan and Levin 2005; Bossu and Near 2009). Despite the limitations of examining only one *H. cyanoguttatus* for our species tree analysis, the nuclear and mitochondrial data clearly support distinct alternative inferences for the evolutionary relationships of *H. cyanoguttatus* and the molariform and papilliform *H. minckleyi* that exhibit “*H. cyanoguttatus*” mitochondrial haplotypes.

The nuclear gene trees also did not support recognition of *H. minckleyi*'s alternative pharyngeal morphotypes as evolutionarily distinct lineages. At best, the species tree reconstructions grouped the molariform and papilliform *H. minckleyi* with the “*H. cyanoguttatus”* cyt*b* haplotype as the two most evolutionarily similar *H. minckleyi* (Fig. 5). The overlapping concordance factors for the putative relationships among the four *H. minckleyi* indicate that the papilliform and molariform pharyngeal morphotypes are not genetically differentiated at most loci (Fig. 5). This corroborates a number of previous allozyme studies that found no differentiation between the *H. minckleyi* morphotypes (Kornfield and Koehn 1975; Sage and Selander 1975; Kornfield et al. 1982). However, because hybridization could be blurring any genetic distinctiveness of the two pharyngeal morphotypes and the morphotypes are not absolutely discrete in their jaw morphology (Fig. 3C), creative ways will be needed to investigate the genetic basis of the alternative morphotypes. For instance, association mapping of wild populations that combine large numbers of genetic markers with quantitative measurements of *H. minckleyi* variation in pharyngeal jaw traits might prove useful in delineating any genetic differences underlying the bimodal jaw morphologies (Gompert et al. 2012).

The low-average nuclear sequence divergence among the *H. minckleyi* and discordance in the mitochondrial and nuclear gene trees are both consistent with biased introgression of the mitochondria (Figs 4 and 5; Table 2). However, without large-scale cloning or phasing of nuclear genes and genotyping nuclear sequences from many more *H. minckleyi*, it is difficult to statistically determine whether the nuclear genes generally exhibit much lower rates of introgression (Mims et al. 2010). Nevertheless, this type of extensive mitochondrial introgression without substantial nuclear introgression is not uncommon. Similar patterns have been documented in animal taxa as disparate as the *Drosophila yakuba* species (Bachtrog et al. 2006), Arctic and Brook charr (Doiron et al. 2002), North American darters (Bossu and Near 2009), and rainforest lizards (Singhai and Moritz 2012). Generally, there are a number of mechanisms that can influence the propensity for the maternally inherited mitochondria to preferentially introgress into a narrowly endemic species like *H. minckleyi*. Biased intrinsic incompatibilities or breakdown in sexual recognition systems could result in asymmetric backcrossing of female *H. cyanoguttatus* with male *H. minckleyi* (Chan and Levin 2005; Chatfield et al. 2010). The presence of *H. cyanoguttatus* mtDNA haplotypes throughout Cuatro Ciénegas could also have resulted from sex-biased female dispersal (Singhai and Moritz 2012). Like many cichlids, male *H. minckleyi* are highly territorial and potentially non-dispersive (Fryer and Iles 1972; Kornfield and Taylor 1983). Alternatively, there could be enhanced selection on the mitochondrial locus due to abiotic factors such as temperature that varies extensively within the many pools and riverine habitats of Cuatro Ciénegas (Minckley 1969; Doiron et al. 2002; Funk and Omland 2003; Tobler and Carson 2010). Advantageously, the replicate nature of the aquatic environments within Cuatro Ciénegas could provide a rigorous geographic framework for teasing apart the importance of intrinsic and extrinsic factors contributing to both biased mitochondrial introgression and hybridization in general within *H. minckleyi*.

The absolute lack of genetic divergence in the introgressed “*H. cyanoguttatus”* cyt*b* also indicates the introgression could be recent (Hulsey et al. 2004). Furthermore, like a number of documented cases of hybridization (Daniels and Corbett 2003; Riley et al. 2003; Streelman et al. 2004), the introgression into *H. minckleyi* could be human mediated. It seems feasible that the ∼ 100-year-old canals that now connect this previously isolated valley with the Rio Grande drainage could have provided a conduit for gene flow between *H. cyanoguttatus* and *H. minckleyi* (Chaves-Campos et al. 2011b). Prior to this connection, Cuatro Ciénegas was completely endorheic and likely had no natural outflow for the last several hundred thousand years (Minckley 1969; Chaves-Campos et al. 2011a). Also, *Herichthys* species tend to produce hundreds of offspring during a single breeding event, breed multiple times a year, and can become reproductively active within about 9 months (Kornfield et al. 1982; Miller et al. 2006). Therefore, during the last century, the man-made alterations to the Cuatro Ciénegas valley coupled with the reproductive life history of these cichlids could easily have facilitated extensive and relatively rapid introgression of *H. cyanoguttatus* genotypes into *H. minckleyi*.

Nevertheless, although hybridization appears to be occurring in this polymorphic species, the trophic variation found within *H. minckleyi* could have no relationship with introgression. The polymorphism could be entirely the result of either phenotypic plasticity or past diversifying selection (Smith and Skulason 1996). The type of bimodal trophic variation found in *H. minckleyi* is environmentally induced in a number of groups (Pfennig and McGee 2010), and many cichlids are known to exhibit phenotypic remodeling in response to eating hard-shelled prey (Greenwood 1965; Meyer 1990; Muschick et al. 2011). Yet, there does appear to be a genetic component to the pharyngeal variation in *H. minckleyi* (Trapani 2003). Isolation of a genetic basis to the heritable differences between the pharyngeal morphotypes could provide solid evidence that plasticity is not wholly responsible for the polymorphism (Sage and Selander 1975). Yet, it is also feasible that the genetic basis of the polymorphism evolved within *H. minckleyi* prior to hybridization with *H. cyanoguttatus* (Valosaari et al. 2008). If a genetic basis to the morphotypes could be isolated and then screened in populations of *H. cyanoguttatus* in combination with detailed quantification of its pharyngeal morphology, one could determine whether the polymorphism originated within Cuatro Ciénegas or alternatively occurred within and subsequently introgressed from *H. cyanoguttatus* populations outside the valley. Determining that introgression is directly responsible for the origin of adaptive traits, even those as relatively discrete as the intraspecific divergence found within *H. minckleyi*, remains challenging (Grant and Grant 2008; Pardo-Diaz et al. 2012).

Introgressive hybridization as documented here will likely continue to receive increased attention as an evolutionary mechanism contributing to adaptive divergence (Seehausen 2004; Mallet 2005; Parnell et al. 2012). There are a number of other trophically polymorphic cichlid species in places like the African Great Lakes and in the crater lakes of Nicaragua that could be examined to determine whether there is a general link between introgressive hybridization and polymorphism (Meyer 1990; Smith and Skulason 1996; Barluenga et al. 2006). However, because most cichlid assemblages contain a large number of recently diverged and frequently sympatric species that could hybridize (Fryer and Iles 1972; Streelman et al. 2004), determining the effect of hybridization on adaptation in most cichlids could be difficult (Mims et al. 2010). In contrast, the substantial independent evolutionary history and minimal number of congeneric species in Cuatro Ciénegas could allow us to determine whether *H. minckleyi* is rapidly diverging along an ecological axis, merging via introgressive hybridization, or maintaining polymorphic jaw variation within the context of substantial interspecific gene flow. Future studies that incorporate an even greater breadth of genomically independent loci coupled with novel analytical techniques should allow us to dissect out how and whether the exceptional variation in *H. minckleyi'*s jaw phenotypes is the result of introgressive hybridization.

## Data Accessibility

DNA sequences: Supplementary Table 2 and Genbank accessions: KC839618-KC839812; JQ425647-JQ425654; JX213216-JX307067; KF145983-KF146149; KG039431-KG039478.

Nexus tree files and morphological data on DRYAD: entry doi: 10.5061/dryad.44v1s.

## Supporting Information

**Table S1.** The 84 primer pairs used in the species tree analyses. All 71 “Min” primers were designed for this study from commercially purchased randomly amplified reads of a single *Herichthys cyanoguttatus*.

**Table S2.** GenBank numbers of the eight *Herichthys* examined for the 84 nuclear loci.
