# Supplementary material for: Introgressive hybridization in a trophically polymorphic cichlid
Source: Ecol Evol. 2013 Oct 18;3(13):4536–47. doi: 10.1002/ece3.841 (PMC3856752; doi:10.1002/ece3.841)
Supplement: Supplementary file 1 [file ece30003-4536-SD1.docx]

**Supplementary Table 1** The 84 primer pairs used in the species tree analyses. All 71 “Min” primers were designed for this study from commercially purchased randomly amplified reads of a single *H. cyanoguttatus.* The remaining 13 primers were obtained from published genetic studies of other cichlids ^1^ Nagl *et al*. 1998, ^2^ Carleton & Kocher 2001, ^3^ Li et al. 2011, ^4^ Hulsey *et al*. 2011. The amplicon sizes from *H. cyanoguttatus* as well as the forward and reverse primer sequences are given.

| Locus | Amplicon Size (bp) | Forward (5' to 3') | Reverse (5' to 3') |
| --- | --- | --- | --- |
| MinD01 | 609 | TTCGCTCTTTGCACATCTTAAC | CGCTCTTACAGGACTCCTCTTC |
| MinD05 | 406 | AAGCTGACAGTGAAGACCTTGTC | GATTTAGTTCTGGCCCTTGAGTT |
| MinE02 | 497 | AGCACAATGGAAGGCAGCTA | ATGATTTGCATGAAACACTCCTT |
| MinE11 | 202 | CCTGCCCACTCCTTACTTATTTA | AACCCTCAAATACCACAGAGGAT |
| MinF01 | 496 | CTGAATTCGTCTAAACACCTGCT | GCCACATTGTTTTGATTTTGTGT |
| MinF12 | 634 | CAGCTGATCGTTATTTTTAGTTCTTG | GCCACATCTTTAGGAAGAGAAGG |
| MinH01 | 498 | TGATTTTGGAAAGGAATGTGTTT | ACCATTATACCCCCGATTACAAC |
| MinH02 | 472 | CTGTTGCAGAGGATTGTATGTCA | GAATTTCAAGCATGCTAGTGACC |
| MinH08 | 283 | CACCTGATCTTATCTCTCCCTCATA | GGCAAAGAGCATAGTGTTTAGGTTA |
| MinH011 | 396 | AAGGGGAGAAGTAGAAGATTAGCAA | GTATGATGCTCTGTAACTCCAGGTT |
| MinP2A01 | 276 | ACACTACAGAGAGCAACACAAACAG | ATGAAAATGTACAAGCAAAGAGGAC |
| MinP2A08 | 395 | GCCATGGCTATTTATAGCTTTTGTA | CTTCATAATCATTCAAGGCAAATTC |
| MinP2A09 | 429 | GTCAGAGTGGAAAAGAAACAGGTAA | AATTAGCTTACCGACAACTAACTGG |
| MinP2A11 | 434 | AGATGAACCATCATATACCCAAGAA | CTGAAAGTCAATGGAAACATGAAC |
| MinP2B09 | 418 | ACTGTGAACTGAGTCCTCTTCCTCT | GTACACTATGGACAGGTAGCCAATC |
| MinP2C11 | 349 | CCACAGAATGTGAAAACAGTGATTA | CGAGTTAACAGGATGAAAAACAAGT |
| MinP2C12 | 458 | TACCACCTCTTTCAGATGACTTACC | TTAAAATGTAAGCAATGGTTTGTTG |
| MinP2C01 | 496 | GCTTGCTTCATATTCCTCATTATCT | TTATGATGAGACTTGAACTTTGCTG |
| MinP2E07 | 365 | CAAGAGGTCGTATGACATATCCAA | TAAAGAAAGCCTGTAAAACATGTGG |
| MinP2E11 | 448 | GGAATTGGAAGACAAGATTGTTATG | AATTGATCAGGTTTTCATCAGAGAG |
| MinP2F07 | 447 | AGTCCTACTCCAAGATCCTTATGGT | ACATCAAGCTGTAATGAAGCTCAG |
| MinP2F10 | 529 | CAGTAGAAACGTATTTTCGAGAACG | ATAAACTGTCTGCGTTACCTAGCAC |
| MinP2G02 | 554 | ATTATTTTATTTACCTTGGGGGTCA | TCTAGAAAAGTAAGCCGTGCTAAAA |
| MinP2G04 | 472 | GGCTGAAACACAAGTTATCATCATT | AGAGAGTGATGTTAGCACAGAAGGT |
| MinP2H06 | 485 | GACTCTGACCCTAAACCTGACTTC | GGTTAAAGTTGTGGCAGATAACAAT |
| MinP2H10 | 311 | GACAAAAGCTATGAAAAGTGATGCT | AAGGAGACATGACTAAAAACAGACG |
| MinP2H12 | 281 | GATACTTCACATAGTCTCGGCAACT | TGATACTGTTGCTACCTGATTTTGA |
| MinP2HO4 | 463 | GACTGATGTTTTCTGGGATTACATT | GAGACTGCAGATCCTTTACACTCTG |
| MinP2H05 | 352 | CACAGGACCTTAGTTCTTTCACTGT | TAAGGAGCTTTCTTGTCTTGATGTC |
| MinP3A02 | 238 | TATAAGACTGTGCAGCCTCTTACCT | CGATGACTCCTTGGTAATAGGAATA |
| MinP3A07 | 552 | AGTGCTGAAAGCTAAATGTCTTGTT | ATTAAGATTTCACCCTATAGCAGGT |
| MinP3A11 | 194 | AGGTTGATTACTGAACACCAATGAT | AGGCTGAGTGAAATCCTCTTTTATT |
| MinP3B04 | 560 | AATTCGTTCCTCACTTTCTGAATTT | ATGGTTTTAAGTATGAACTGCCACT |
| MinP3B06 | 488 | AGTCTTCAAGAGAGAAGCTTGTGAA | TAACATTAGCCTGCTTCTTAAACCA |
| MinP3C05 | 426 | AACATAGTGCAGAGAAACTTCGATT | CTCACTGAAAGCTCCTTTACATTTC |
| MinP3C08 | 562 | ACATGTAAAATCAGGATTCAGCTTC | CATCACACTGCTAAGACAAATGAGT |
| MinP3C09 | 421 | CCCTAACTCCTAAAACAATCTGTCA | AGCTCATTGTAGAGTCTCAGCAGTT |
| MinP3C12 | 439 | ATCAAGAGAACACCCAATTAGACTG | GTTTTCCAGCATGTTCTTCCTACTA |
| MinP3D06 | 421 | CATCCTTTGTCCAATATATCCACTC | CCAAGTTATAAGATCAGATCACAACC |
| MinP3D07 | 466 | TTTGGAGTAGGCTAGCACTTTCTTA | ATGTAGCCCTAATACCCCTTCATAG |
| MinP3E02 | 495 | ATCTCATGAAGCTCAAGCACTAAAC | CTAACTGGGACCAGAATAAAACAAA |
| MinP3E05 | 391 | GTGCGAGAAGCTGAGAAATACTCTA | ACTTCATGAGTGACCAGAAAAACTT |
| MinP3E08 | 455 | GTTAGGAAGGGGTTGAAGAAGAAGT | AGTCTGTTATCAACCTGCTACCTGT |
| MinP3E10 | 488 | AATAATGGCTTTGTGTGGTTGTATC | TGATAGACAGGGAATAGGCTCAATA |
| MinP3F01 | 512 | GCAAACAACAGTTTCCTCTGTCC | TGAGCATAAACATTTGTGAGGTAAA |
| MinP3F09 | 460 | TTACTTTCCTTCCTCATTCACTCAC | ATAAATGGGGAGGGTTGCATTAG |
| MinP3G07 | 419 | CCTAGGAGCATAGTGTGGAGTAGG | CTTTCTTTAAAAGCAGCCTTCCTC |
| MinP3G11 | 447 | GATTGTGGTGAAGTATACTGCTGTG | GATCAGATGACTCTGTTATGCACTG |
| MinP3GO5 | 447 | ATGCCACATAATGTTCAAATTTCTT | ATTTCAAGGTTACACACTGTTTTCC |
| MinP4A03 | 217 | CTGTTTTATCTTTTAGGGCACTGAC | ATCAATTAGTTAAGCTGCAGGAATG |
| MinP4A06 | 469 | TTTGGCTTTCATATACAAACAATGA | GTTACATCACAGCAGGTCTCACTAC |
| MinP4A09 | 345 | GTCTGGTCTTCAATCATCCGAAA | TTTACTTTATTACATTTGGCAGACG |
| MinP4A11 | 507 | GTGTCAGTCACTACCAGCTACAGAT | AATTCATTCACACATTGATTCACAG |
| MinP4A12 | 427 | GGAAATAGGTTGTGATGGTCCTAGA | CCATAGGAGTAGGGAAAGGGTATTA |
| MinP4B04 | 321 | CCAAGTTTCTGGAGGTAGTCTCTAA | TTACTTTGGTAGTCTTCCTGCTTGT |
| MinP4B12 | 324 | ATTTTTAAAGGTAGGATTGGGCTTC | ACAAATCTGCGTTTGTAGTTCCTT |
| MinP4C08 | 462 | TGGACAATGTTAGTCAAGTATGAGAA | TTGAATGGGGTACATCAGATAACTT |
| MinP4C10 | 366 | CGATTACTTTTGATCCACTGAAAAT | AGACTTTGGAAAAGTTTCCCTCTTA |
| MinP4C12 | 358 | GAATGGTTGAAAAAGAAAAGAATCA | TGAGCATACAGTGTGAGGCTTATAG |
| MinP4CO1 | 426 | TCCATAGTTTTTAGGCCACTGATAG | ACTGCGTTCAGCTACTTTAGCATAC |
| MinP4D01 | 126 | AGGATTACCAAACCACTGATGATAA | GTAGCTGGGCTTTAAATTATCCAGT |
| MinP4D06 | 374 | GAAATATCCTGGTCTCAGAGTGATG | CTGTGTCTCAACACAACATATCACA |
| MinP4D09 | 146 | ACAGCACACACCTAATATCATTCCT | AGCAACATGCATATAACTCAAACAA |
| MinP4D12 | 240 | ATTTGCAATAGCTTGTGAAAAGATT | ACGTCAATATCCACTTAAACCAGAC |
| MinP4E01 | 336 | CTGGGTGATCTCAGATGTTCTTACT | CAAAGAAACTTTTCTGAGGCAGTAG |
| MinP4E03 | 295 | GACATTCAATCATCAATCAATCAAA | AGAGACTGCCAGTGTTTTCAAATTA |
| MinP4E07 | 328 | ACATATCCATAGTGAGTGTGCTCCT | AATAATAGCTCACTGGCTAACATGC |
| MinP4E09 | 206 | CTTTAAACTCAGATCGTCCTCTCAC | GTTTTGAGAGGCAAGTAAAACACAC |
| MinP4E10 | 419 | TCATGTAGGTATCAGTTCATTGAGG | TTTGCAGTGTTCTTCTTCTTCTTTT |
| MinP4G01 | 436 | AAACTGTCCTGTTACTGCCAAAGTA | TCGTTAGTTTATGAGGTTTTTCTGC |
| Throb^4^ | 957 | GACGACCCGTACAGYCCCGCCTA | TGTACTCAGTTCTGATGGCTGAGGG |
| MSX^4^ | 951 | ATGGCCCCATCGAYCGCCATGTC | AGGTGGTACATRCTGTATCC |
| NaglA^1^ | 625 | TACGCCAACAATGTGCTCTCC | GCATGGTTCAGTGGTGGTTTT |
| Rag1^3^ | 970 | GCCGCCAGATCTTCCAGCCCT | TGCGGGCGTAGTTTCCATTCA |
| Rhodcic^1^ | 708 | AGCTTATGCTGCTCTGGGTGCC | TCCAAATTCAGAGCGTTGATG |
| Sreb2^3^ | 940 | ATGGCGAACTAYAGCCATGC | CTGGATTTTCTGCAGTASAGGAG |
| SH3PX3^3^ | 589 | GACGTTCCCATGATGGCWAAAT | CATCTCYCCGATGTTCTCGTA |
| Somat^4^ | 611 | TACGTCATCCTGCGCTSCG | GAAGGCGTAGSGGATGGGGTT |
| Tbr1^3^ | 606 | TGTCTACACAGGCTGCGACA | GATGTCCTTRGWGCAGTTTTT |
| Wnt7b^4^ | 519 | CCTCTCCATGCAAGTCTGGT | CCAACTACTGCGAGGAGGAC |
| Dystb^4^ | 549 | GCGCATTGCAGACTTTGATCT | TGCTGCTGTTKCCAGATGCCTAAT |
| Enc^3^ | 763 | ATGCTGGAGTTTCAGGACAT | AGCMACTGGGTCAAACTGCTC |
| Glyt^3^ | 766 | GACTGTCMAAGATGACCACMT | CCCAAGAGGTTCTTGTTRAAGAT |
| LWS^2^ | 730 | CTGTGCTACCTT GCTGTGTGG | TG GCCATCCGTGCTGTTGCC |
