# Supplementary material for: Introgressive hybridization in a trophically polymorphic cichlid
Source: Ecol Evol. 2013 Oct 18;3(13):4536–47. doi: 10.1002/ece3.841 (PMC3856752; doi:10.1002/ece3.841)
Supplement: Supplementary file 2 [file ece30003-4536-SD2.docx]

**Supplementary Table 2** Genbank numbers of the eight *Herichthys* examined for the 84 nuclear loci. The molariform (M) and papilliform (P) *H. minckleyi* either exhibited haplotype III from the Churince collection site or the “*H. cyanoguttatus*” (C) mitochochondrial haplotype from the same location and the haplotype is indicated with a superscripted abbreviation. The abbreviations for each individual are as follows: *H. minckleyi* (P^III^). *H. minckleyi* (M^C^), *H. minckleyi* (P^C^), *H. minckleyi* (M^III^), *H. deppii* (D), *H. cyanoguttatus* (C), *H. carpintes* (R), and *H. tamasopoensis* (T).

| Locus | P^III^ | P^C^ | M^C^ | M^III^ | D | C | R | T |
| --- | --- | --- | --- | --- | --- | --- | --- | --- |
| MinD01 | KG039443 | KG039449 | KG039431 | KG039437 | KG039473 | KG039461 | KG039455 | KG039467 |
| MinD05 | JX306778 | JX306779 | JX306776 | JX306777 | JX306783 | JX306781 | JX306780 | JX306782 |
| MinE02 | KF146000 | KF146001 | KF145998 | KF145999 | KF146005 | KF146003 | KF146002 | KF146004 |
| MinE11 | KF145992 | KF145993 | KF145990 | KF145991 | KF145997 | KF145995 | KF145994 | KF145996 |
| MinF01 | JX262808 | JX262809 | JX262806 | JX262807 | JX262813 | JX262811 | JX262810 | JX262812 |
| MinF12 | KF146008 | KF146009 | KF146006 | KF146007 | KF146013 | KF146011 | KF146010 | KF146012 |
| MinH011 | JX254890 | JX254891 | JX254888 | JX254889 | JX254895 | JX254893 | JX254892 | JX254894 |
| MinH01 | JX262816 | JX262817 | JX262814 | JX262815 | JX262820 | JX262818 | JX262819 | JX262819 |
| MinH02 | JX262823 | JX262824 | JX262821 | JX262822 | JX262828 | JX262826 | JX262825 | JX262827 |
| MinH08 | JX290102 | JX290103 | JX290100 | JX290101 | JX290107 | JX290105 | JX290104 | JX290106 |
| MinP2A01 | KF146016 | KF146017 | KF146014 | KF146015 | KF146021 | KF146019 | KF146018 | KF146020 |
| MinP2A08 | JX262831 | JX262832 | JX262829 | JX262830 | JX262836 | JX262834 | JX262833 | JX262835 |
| MinP2A09 | JX238463 | JX238464 | JX238461 | JX238462 | JX238468 | JX238466 | JX238465 | JX238467 |
| MinP2A11 | JX306922 | JX306923 | JX306920 | JX306921 | JX306927 | JX306925 | JX306924 | JX306926 |
| MinP2B09 | JX262839 | JX262840 | JX262837 | JX262838 | JX262844 | JX262842 | JX262841 | JX262843 |
| MinP2C11 | JX262847 | JX262848 | JX262845 | JX262846 | JX262852 | JX262850 | JX262849 | JX262851 |
| MinP2C12 | KF146024 | KF146025 | KF146022 | KF146023 | KF146029 | KF146027 | KF146026 | KF146028 |
| MinP2C01 | KF146032 | KF146033 | KF146030 | KF146031 | KF146037 | KF146035 | KF146034 | KF146036 |
| MinP2E07 | JX290110 | JX290111 | JX290108 | JX290109 | JX290115 | JX290113 | JX290112 | JX290114 |
| MinP2E11 | JX306786 | JX306787 | JX306784 | JX306785 | JX306791 | JX306789 | JX306788 | JX306790 |
| MinP2F07 | JX306834 | JX306835 | JX306832 | JX306833 | JX306839 | JX306837 | JX306836 | JX306838 |
| MinP2F10 | KF146040 | KF146041 | KF146038 | KF146039 | KF146045 | KF146043 | KF146042 | KF146044 |
| MinP2G02 | KF146048 | KF146049 | KF146046 | KF146047 | KF146053 | KF146051 | KF146050 | KF146052 |
| MinP2G04 | KG039444 | KG039450 | KG039432 | KG039438 | KG039468 | KG039462 | KG039456 | KG039474 |
| MinP2HO4 | KF146056 | KF146057 | KF146054 | KF146055 | KF146061 | KF146059 | KF146058 | KF146060 |
| MinP2H05 | KF146064 | KF146065 | KF146062 | KF146063 | KF146069 | KF146067 | KF146066 | KF146068 |
| MinP2H06 | JX306930 | JX306931 | JX306928 | JX306929 | JX306935 | JX306933 | JX306932 | JX306934 |
| MinP2H10 | JX306842 | JX306843 | JX306840 | JX306841 | JX306847 | JX306845 | JX306844 | JX306846 |
| MinP2H12 | JX306938 | JX306939 | JX306936 | JX306937 | JX306943 | JX306941 | JX306940 | JX306942 |
| MinP3A02 | JX306946 | JX306947 | JX306944 | JX306945 | JX306951 | JX306949 | JX306948 | JX306950 |
| MinP3A07 | JX306850 | JX306851 | JX306848 | JX306849 | JX306855 | JX306853 | JX306852 | JX306854 |
| MinP3A11 | JX306986 | JX306987 | JX306984 | JX306985 | JX306991 | JX306989 | JX306988 | JX306990 |
| MinP3B04 | JX306858 | JX306859 | JX306856 | JX306857 | JX306863 | JX306861 | JX306860 | JX306862 |
| MinP3B06 | JX306954 | JX306955 | JX306952 | JX306953 | JX306959 | JX306957 | JX306956 | JX306958 |
| MinP3C05 | KF146072 | KF146073 | KF146070 | KF146071 | KF146077 | KF146075 | KF146074 | KF146076 |
| MinP3C08 | KF146080 | KF146081 | KF146078 | KF146079 | KF146085 | KF146083 | KF146082 | KF146084 |
| MinP3C09 | JX306962 | JX306963 | JX306960 | JX306961 | JX306967 | JX306965 | JX306964 | JX306966 |
| MinP3C12 | KF146088 | KF146089 | KF146086 | KF146087 | KF146093 | KF146091 | KF146090 | KF146092 |
| MinP3D06 | JX306970 | JX306971 | JX306968 | JX306969 | JX306975 | JX306973 | JX306972 | JX306974 |
| MinP3D07 | JX306866 | JX306867 | JX306864 | JX306865 | JX306871 | JX306869 | JX306868 | JX306870 |
| MinP3E02 | JX306981 | JX306980 | JX306983 | JX306982 | JX306976 | JX306978 | JX306979 | JX306977 |
| MinP3E05 | JX306794 | JX306795 | JX306792 | JX306793 | JX306799 | JX306797 | JX306796 | JX306798 |
| MinP3E08 | JX306994 | JX306995 | JX306992 | JX306993 | JX306999 | JX306997 | JX306996 | JX306998 |
| MinP3E10 | JX307002 | JX307003 | JX307000 | JX307001 | JX307007 | JX307005 | JX307004 | JX307006 |
| MinP3F01 | JX306874 | JX306875 | JX306872 | JX306873 | JX306879 | JX306877 | JX306876 | JX306878 |
| MinP3F09 | JX307010 | JX307011 | JX307008 | JX307009 | JX307015 | JX307013 | JX307012 | JX307014 |
| MinP3G05 | JX306882 | JX306883 | JX306880 | JX306881 | JX306887 | JX306885 | JX306884 | JX306886 |
| MinP3G07 | JX306802 | JX306803 | JX306800 | JX306801 | JX306807 | JX306805 | JX306804 | JX306806 |
| MinP3G11 | JX262855 | JX262856 | JX262853 | JX262854 | JX262860 | JX262858 | JX262857 | JX262859 |
| MinP4A03 | JX307018 | JX307019 | JX307016 | JX307017 | JX307023 | JX307021 | JX307020 | JX307022 |
| MinP4A06 | JX307026 | JX307027 | JX307024 | JX307025 | JX307031 | JX307029 | JX307028 | JX307030 |
| MinP4A09 | JX307034 | JX307035 | JX307032 | JX307033 | JX307039 | JX307037 | JX307036 | JX307038 |
| MinP4A11 | JX306890 | JX306891 | JX306888 | JX306889 | JX306895 | JX306893 | JX306892 | JX306894 |
| MinP4A12 | JX306898 | JX306899 | JX306896 | JX306897 | JX306903 | JX306901 | JX306900 | JX306902 |
| MinP4B04 | JX307042 | JX307043 | JX307040 | JX307041 | JX307047 | JX307045 | JX307044 | JX307046 |
| MinP4B12 | JX306810 | JX306811 | JX306808 | JX306809 | JX306815 | JX306813 | JX306812 | JX306814 |
| MinP4C01 | JX262863 | JX262864 | JX262861 | JX262862 | JX262868 | JX262866 | JX262865 | JX262867 |
| MinP4C08 | JX307050 | JX307051 | JX307048 | JX307049 | JX307055 | JX307053 | JX307052 | JX307054 |
| MinP4C10 | JX307058 | JX307059 | JX307056 | JX307057 | JX307063 | JX307061 | JX307060 | JX307062 |
| MinP4C12 | JX307066 | JX307067 | JX307064 | JX307065 | JX307071 | JX307069 | JX307068 | JX307070 |
| MinP4D01 | KG039445 | KG039451 | KG039433 | KG039439 | KG039475 | KG039463 | KG039457 | KG039469 |
| MinP4D06 | KF146096 | KF146097 | KF146094 | KF146095 | KF146101 | KF146099 | KF146098 | KF146100 |
| MinP4D09 | KF146104 | KF146105 | KF146102 | KF146103 | KF146109 | KF146107 | KF146106 | KF146108 |
| MinP4D12 | JX306906 | JX306907 | JX306904 | JX306905 | JX306911 | JX306909 | JX306908 | JX306910 |
| MinP4E01 | KG039446 | KG039452 | KG039434 | KG039440 | KG039476 | KG039464 | KG039458 | KG039470 |
| MinP4E03 | KG039447 | KG039453 | KG039435 | KG039441 | KG039477 | KG039465 | KG039459 | KG039471 |
| MinP4E07 | JX306818 | JX306819 | JX306816 | JX306817 | JX306823 | JX306821 | JX306820 | JX306822 |
| MinP4E09 | KG039448 | KG039454 | KG039436 | KG039442 | KG039478 | KG039466 | KG039460 | KG039472 |
| MinP4E10 | KF146112 | KF146113 | KF146110 | KF146111 | KF146117 | KF146115 | KF146114 | KF146116 |
| MinP4G01 | JX306826 | JX306827 | JX306824 | JX306825 | JX306831 | JX306829 | JX306828 | JX306830 |
| Throb | KF146120 | KF146121 | KF146118 | KF146119 | KF146125 | KF146123 | KF146122 | KF146124 |
| MSX | JQ425651 | JQ425653 | JQ425652 | JQ425654 | JQ425649 | JQ425648 | JQ425647 | JQ425650 |
| NaglA | KF146131 | KF146133 | KF146132 | KF146130 | KF146129 | KF146127 | KF146126 | KF146128 |
| Rag1 | JX213232 | JX213233 | JX213230 | JX213229 | JX213231 | JX213234 | JX192593 | JX213235 |
| Rhodcic | KF146128 | KF146129 | KF146126 | KF146127 | KF146133 | KF146131 | KF146130 | KF146132 |
| Sreb2 | JX213241 | JX192594 | JX213240 | JX213242 | JX213238 | JX213239 | JX213237 | JX213248 |
| SH3PX3 | JX192595 | JX213244 | JX213243 | JX213245 | JX213247 | JX213246 | JX213249 | JX213250 |
| Somat | KF146136 | KF146137 | KF146134 | KF146135 | KF146141 | KF146139 | KF146138 | KF146140 |
| Tbr1 | JX192590 | JX213251 | JX213250 | JX213252 | JX213254 | JX213256 | JX213253 | JX213255 |
| Wnt7b | KF146144 | KF146145 | KF146142 | KF146143 | KF146149 | KF146147 | KF146146 | KF146148 |
| Dystb | KF145988 | KF145989 | KF145987 | KF145985 | KF145983 | KF145984 | KF145986 | KF145982 |
| Enc | JX213219 | JX213220 | JX213218 | JX213217 | JX213216 | JX213222 | JX213221 | JX192591 |
| Glyt | JX192592 | JX213226 | JX213227 | JX213225 | JX213223 | JX213228 | JX213224 | JX213224 |
